# Performance of blink reflex in patients during anesthesia induction with propofol and remifentanil: prediction probabilities and multinomial logistic analysis

**DOI:** 10.1186/s12938-020-00828-6

**Published:** 2020-11-14

**Authors:** Ana Leitão Ferreira, Catarina S. Nunes, Sérgio Vide, João Felgueiras, Márcio Cardoso, Pedro Amorim, Joaquim Mendes

**Affiliations:** 1 LAETA, INEGI, Faculdade de Engenharia, Universidade do Porto, Porto, Portugal; 2 Centro de Investigação Clínica em Anestesiologia, Serviço de Anestesiologia, Centro Hospitalar do Porto, Largo Professor Abel Salazar, 4099-001 Porto, Portugal; 3 Departamento de Ciências e Tecnologia, Universidade Aberta, Delegação do Porto, Porto, Portugal; 4 Departamento de Anestesia, Unidade Local de Saúde de Matosinhos, Hospital Pedro Hispano, Matosinhos, Portugal; 5 Serviço de Neurofisiologia, Centro Hospitalar do Porto, Porto, Portugal

**Keywords:** Blink reflex, Loss of responsiveness, Anesthesia monitoring, Prediction, Propofol, Sedation

## Abstract

**Background:**

The amount of propofol needed to induce loss of responsiveness varied widely among patients, and they usually required less than the initial dose recommended by the drug package inserts. Identifying precisely the moment of loss of responsiveness will determine the amount of propofol each patient needs. Currently, methods to decide the exact moment of loss of responsiveness are based on subjective analysis, and the monitors that use objective methods fail in precision. Based on previous studies, we believe that the blink reflex can be useful to characterize, more objectively, the transition from responsiveness to unresponsiveness. The purpose of this study is to investigate the relation between the electrically evoked blink reflex and the level of sedation/anesthesia measured with an adapted version of the Richmond Agitation–Sedation Scale, during the induction phase of general anesthesia with propofol and remifentanil. Adding the blink reflex to other variables may allow a more objective assessment of the exact moment of loss of responsiveness and a more personalized approach to anesthesia induction.

**Results:**

The electromyographic-derived features proved to be good predictors to estimate the different levels of sedation/anesthesia. The results of the multinomial analysis showed a reasonable performance of the model, explaining almost 70% of the adapted Richmond Agitation–Sedation Scale variance. The overall predictive accuracy for the model was 73.6%, suggesting that it is useful to predict loss of responsiveness.

**Conclusions:**

Our developed model was based on the information of the electromyographic-derived features from the blink reflex responses. It was able to predict the drug effect in patients undergoing general anesthesia, which can be helpful for the anesthesiologists to reduce the overwhelming variability observed between patients and avoid many cases of overdosing and associated risks. Despite this, future research is needed to account for variabilities in the clinical response of the patients and with the interactions between propofol and remifentanil. Nevertheless, a method that could allow for an automatic prediction/detection of loss of responsiveness is a step forward for personalized medicine.

## Background

In clinical practice, anesthesiologists use a variety of anesthetic drugs during surgery to render the patient unconscious/unresponsive, including the most widely used, intravenous drug propofol. In a previously published work [1,2], we found that the amount of propofol needed to induce loss of responsiveness (LORP) varied widely among patients (~ 300%) and that more than two-thirds of the patients required less than the initial dose recommended by the drug package inserts.

Identifying precisely the moment of LORP during the induction phase of general anesthesia is of extreme importance for the determination of the amount of propofol each patient needs. Using that information will help to guide the drug infusion rate required to maintain an adequate level of anesthesia throughout the surgery [3–5].

Currently, methods to decide the exact moment of LORP are based on subjective analysis [6]. Objectively, there are depth of anesthesia monitors, such as the Bispectral Index (BIS) (Aspect Medical System, Newton, MA, USA), which allow the maintenance of a steady-state during surgery, but do not enable the determination of the instant at LORP, which remains an open issue. BIS evaluation is characterized by a delay following the acquisition of a new dataset, which may even exceed one minute [7]. To prevent complications, such as awareness [8, 9] or excessive anesthesia, the anesthesiologists should be aware of the conditions that cause incorrect BIS readings.

In a previous study, Mourisse et al. [10] found that the components of the blink reflex are attenuated and abolished with increasing concentrations of propofol. Mourisse et al. [11] showed, in another study that the blink reflex was more sensitive than BIS. Their results suggested that the differential sensitivity of the blink reflex components could be useful to monitor the depth of sedation/anesthesia, and thus, to detect when LORP occurs. However, their method used a 10-min stepwise increment in propofol, which is not compatible with anesthesia induction in a surgical setup.

We hypothesize that the blink reflex can be useful to characterize, more objectively, the transition from responsiveness to unresponsiveness during the induction of anesthesia with propofol and remifentanil. The administration of propofol may be stopped at this transition, personalizing the amount of propofol each patient requires and reducing the events of over and underdosing. Then, this can be used to titrate the infusion rate of propofol and to maintain an adequate level of sedation/anesthesia. In the current study, and based on a constant infusion of propofol at a slow rate, we intend to investigate the relationship between the electrically evoked blink reflex and the level of sedation/anesthesia. For this purpose, we extracted different electromyogram (EMG) features, and we compared the ability of these features to distinguish between different levels of sedation/anesthesia. The comparison was carried out using prediction probability analysis and multinomial logistic modeling. Adding the blink reflex to other variables already recorded during general anesthesia may allow a more objective assessment of the exact moment of LORP and a more personalized approach to anesthesia induction.

## Results

Twenty-five patients (16 female and 9 male), aged 61 ± 13, weighing 72 ± 11 kg, heighten 162 ± 9 cm, 1 ASA I, 18 ASA II, 6 ASA III were enrolled. The supraorbital nerve was stimulated with 25.7 ± 8 mA. All patients reached the end of the depth of sedation/anesthesia scale. No patient had hemodynamic or respiratory problems.

Visual inspection of the raw electromyogram and analysis of the signal quality was performed before extracting the features to eventually discard poor-quality electromyogram. No signal was discarded.

**Fig. 1:**
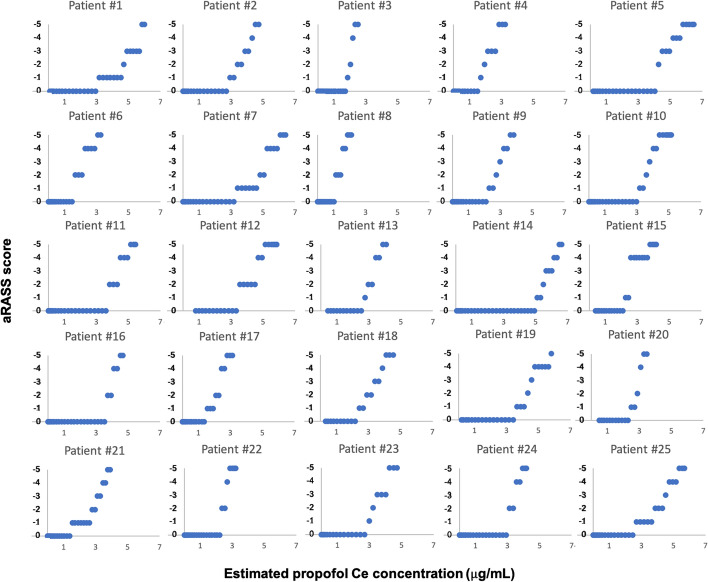
The predicted propofol effect-site concentration vs adapted Richmond Agitation–Sedation Scale (aRASS)

The raw data from the study are presented in Fig. 1; each patient’s observed adapted Richmond Agitation–Sedation Scale (aRASS) scores are plotted against the corresponding predicted propofol effect-site concentrations.

Concerning the raw electromyographic findings, after the propofol infusion was started, *R*_1_ and *R*_2_ decreased gradually. Ce concentrations, aRASS values, and times at which LOR_2_, LOR_1_ and LORP occurred are presented in Table 1. *R*_2_ was the first component to be abolished, followed by the R_1_ component. aRASS median [minimum, maximum] for LOR_2_ was 0 [0, − 2] and for LOR_1_ was − 2 [0, − 4]. All patients were responsive (aRASS < − 5) when the *R*_2_ and *R*_1_ responses were last seen. There was a statistically significant difference between the propofol Ce concentration at LORP and LOR_1_ (*p* < 0.05). The times for endpoints LOR_2_, LOR_1_ and LORP are all statistically different from each other (*p* < 0.001).

**Table 1:** Estimated effect-site concentration of propofol, adapted Richmond Agitation–Sedation Scale values, and times at which LOR_2_, LOR_1_ and LORP occurred. Results are mean ± standard deviation or median [minimum, maximum]

	LOR_2_	LOR_1_	LORP
Time since propofol started (s)	64.70 ± 23.72	112.16 ± 30.82	147.84 ± 27.36
Estimated propofol Ce concentration (µg/mL)	1.45 ± 0.85	2.99 ± 1.19	4.22 ± 1.24
aRASS score	0 [0, − 2]	− 2 [0, − 4]	− 5

*LOR*_*2*_  loss of R_2_ component, *LOR*_*1*_ loss of R_1_ component, *LORP* loss of responsiveness, *aRASS* adapted Richmond Agitation–Sedation Scale, *Ce* effect-site concentration

The time between LORP and LOR_1_ was 35.68 ± 23.41 s. The amount of propofol given between LOR_1_ and LORP was 1.23 ± 0.82 μg/mL.

Results of the features extracted from the EMG signals in the time and frequency domain are presented in Table 2.

**Table 2:** Values from the extracted features during two different time windows: *P*_1_—from 10 to 25 ms, in which it is expected *R*_1_ to be analyzed; *P*_2_—from 25 to 200 ms, in which *R*_2_ is expected to be analyzed

Parameter	Minimum	Maximum	Mean	Standard deviation	Median
*V*_mean_	0.190	0.721	0.443	0.126	0.445
*V*_diff_	0.280	0.840	0.556	0.126	0.555
*P*_mean_ (dB)	0.166	15.162	2.660	2.879	1.555
*P*_max_ (V)	3.770	436.561	68.613	78.169	37.462
*f*_mean_ (Hz)	103.648	203.160	123.763	18.525	116.347
*P*_fmean_ (V)	2.793	314.897	50.984	57.029	28.624
*f*_median_ (Hz)	89.179	168.352	105.909	16.692	98.161
*P*_band_ (dB)	596.219	62,514.798	10,151.828	11,349.090	5730.088
*P*_total_ (dB)	836.969	76,399.833	13,403.153	14,506.321	7834.622
*P*_total/_f_mean_	7.190	845.757	132.339	151.067	71.768
SNR	− 11.314	34.838	− 6.980	2.886	− 7.847
*P*_bandwidth_ (dB)	157.941	310.036	188.047	33.105	172.167
Spectral entropy	0.412	0.546	0.445	0.026	0.436
*V*_mean_	0.130	0.575	0.320	0.088	0.310
*V*_diff_	0.425	0.870	0.680	0.088	0.690
*P*_mean_ (dB)	0.007	14.787	0.509	1.035	0.184
*P*_max_ (V)	0.363	868.099	24.534	52.506	9.724
*f*_mean_ (Hz)	23.459	181.467	58.488	30.876	48.865
*P*_fmean_ (V)	0.169	868.099	21.972	50.663	8.263
*f*_median_ (Hz)	22.419	167.057	46.844	26.412	38.396
*P*_band_ (dB)	32.975	73,225.083	2337.29	4792.514	875.481
*P*_total_ (dB)	35.038	74,511.906	25,660.961	5216.244	926.853
*P*_total_/f_mean_	0.859	2736.873	63.886	156.724	23.885
SNR	− 8.038	44.143	8.691	11.550	4.456
*P*_bandwidth_ (dB)	37.304	250.824	66.134	33.175	53.062
Spectral entropy	0.180	0.496	0.299	0.072	0.278

*V*_mean_ mean amplitude, *V*_diff_  difference between maximum and mean amplitude, *P*_mean_ mean power, *P*_max_ maximum power, *f*_mean_ mean frequency, *P*_meanfrequency_ power at mean frequency, *f*_median_  median frequency, *P*_band_ power band, *P*_total_ total integrated of the spectrum, *P*_total_*/f*_median_ ratio between total power and median frequency, *SNR* signal-to-noise ratio, *P*_bandwidth_ power bandwidth

### Prediction probability and Spearman correlation coefficient

**Fig. 2:**
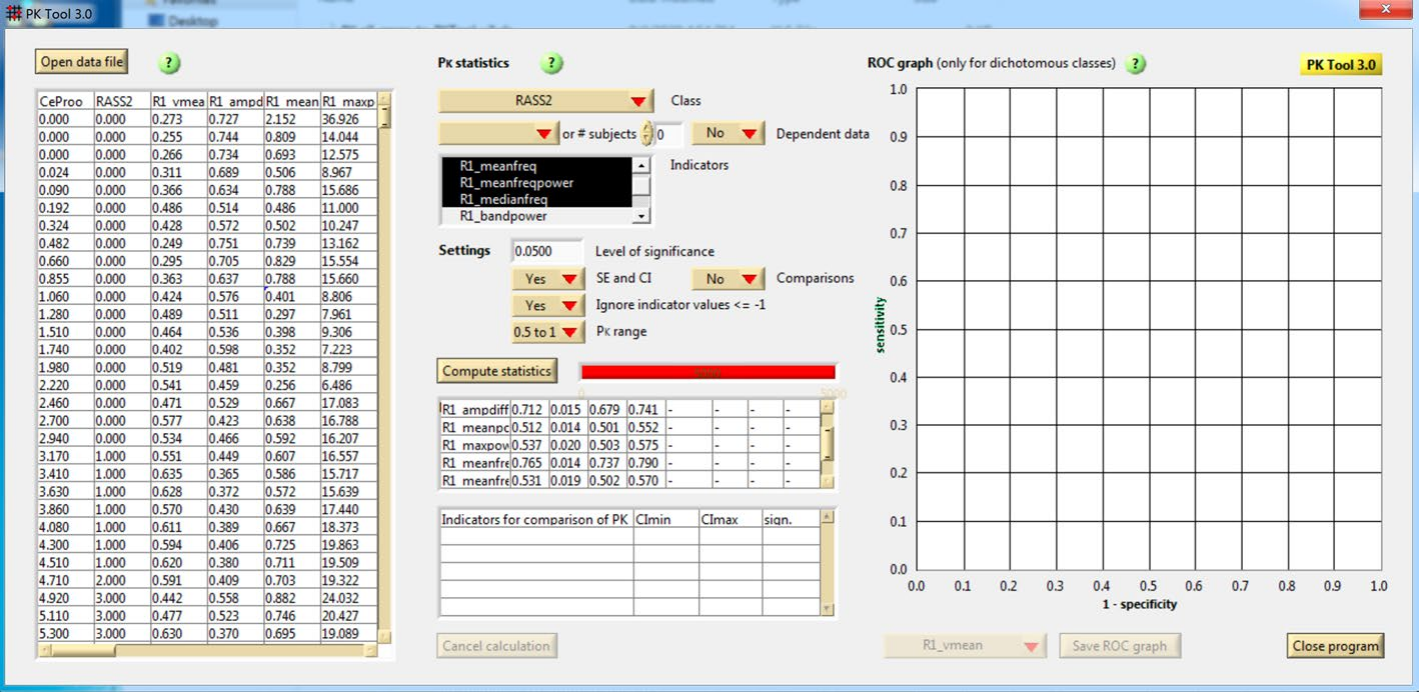
User interface of the program “*P*_*k*_ Tool” for computing prediction probability (*P*_*k*_). Left panel refers to the data reading function. In the middle panel, above, is the class and indicators/features selection such as settings for computation and below, is the calculation and result output unit. Right panel indicates the receiver operating characteristic curve (ROC) only used for dichotomous classes (which is not the case). Results are saved as data sheets

The capacity of all extracted features to adequately assess the clinical sedation/anesthetic depth was evaluated by *P*_*k*_ analyses. The user interface is shown in Fig. 2. The calculated *P*_*k*_ values and correlation coefficients of the electromyography **(**EMG) extracted features are shown in Table 3. *P*_*k*_ values were higher (*P*_*k*_ > 0.700) in the period *T*_1_ for *V*_mean_, *V*_diff_, *f*_mean_, *f*_median_, *P*_bandwidth_, and spectral entropy; and in the period *T*_2_ for *f*_mean_, and spectral entropy. Results indicated that during the period *T*_1_ there was a significant negative association, defined by a correlation coefficient *R* higher than − 0.500 and a *p* < 0.05, between aRASS and *f*_mean_, *f*_median_, and spectral entropy. During the period *T*_1_ there was a significant negative association between propofol Ce concentration and the following features: *f*_mean_, *f*_median_, and spectral entropy. There was no significant correlation between aRASS and the extracted features, and no significant association between the Ce concentration of propofol and the extracted features.

**Table 3:** Prediction probability (P_k_) values calculated between the adapted Richmond Agitation–Sedation Scale (aRASS) and the electromyography (EMG) extracted feature. Correlation coefficient *R* with aRASS and with propofol effect-site (Ce) concentration. *T*_1_ and *T*_2_ are time windows from 10 to 25 ms, and from 25 to 200 ms, respectively

Parameter		aRASS		Correlation coefficient R with aRASS	Correlation coefficient R with propofol Ce concentration
		*P*_*k*_	SE		
T_1_	*V*_mean_	0.712	0.015	0.420**	0.471**
	*V*_diff_	0.712	0.015	− 0.420**	− 0.471**
	*P*_mean_	0.512	0.014	0.025	− 0.064**
	*P*_max_	0.537	0.020	0.073	− 0.010
	*f*_mean_	0.765	0.014	− 0.522**	− 0.522**
	*P*_fmean_	0.531	0.019	0.062	− 0.022
	*f*_median_	0.758	0.014	− 0.507**	− 0.504**
	*P*_band_	0.529	0.019	0.058	− 0.027
	*P*_total_	0.512	0.014	0.025	− 0.064
	*P*_total_/f_median_	0.538	0.020	0.075*	− 0.007
	SNR	0.643	0.071	− 0.114**	− 0.052
	*P*_bandwidth_	0.748	0.014	− 0.487**	− 0.482**
	Spectral entropy	0.771	0.013	− 0.534**	− 0.535**
T_2_	*V*_mean_	0.619	0.018	0.236**	0.201**
	*V*_diff_	0.619	0.018	− 0.236**	− 0.201**
	*P*_mean_	0.610	0.019	− 0.216**	− 0.430**
	*P*_max_	0.578	0.019	− 0.153**	− 0.369**
	*f*_mean_	0.721	0.015	− 0.437**	− 0.415**
	*P*_fmean_	0.538	0.019	− 0.064	− 0.280**
	*f*_median_	0.677	0.016	− 0.353**	− 0.334**
	*P*_band_	0.599	0.018	− 0.194**	− 0.416**
	*P*_total_	0.610	0.018	− 0.216**	− 0.438**
	*P*_total_/f_median_	0.557	0.019	− 0.111**	− 0.325**
	SNR	0.679	0.020	− 0.387**	− 0.452**
	*P*_bandwidth_	0.605	0.017	− 0.211**	− 0.163**
	Spectral entropy	0.745	0.014	− 0.485**	− 0.464**

Prediction probability (*P*_*k*_) values calculated with pooled data from all patients (*n* = 25). The standard error (SE) is also shown. Rank correlation coefficient from pooled data of all patients (n = 25) is shown. *Significant at the 0.05 level. **Significant at the 0.001 level. *V*_mean_ mean amplitude, *V*_diff_ difference between maximum and mean amplitude, *P*_mean_ mean power, *P*_max_ maximum power, *f*_mean_ mean frequency, *P*_meanfrequency_ power at mean frequency, *f*_median_ median frequency, *P*_band_ power band, *P*_*total*_ total integrated of the spectrum, *P*_total_/*f*_median_ ratio between total power and median frequency, SNR  signal-to-noise ratio, *P*_bandwidth_ power bandwidth

The correlation between aRASS and the propofol Ce concentration was given by *P*_*k*_ = 0.886, SE = 0.007 and by *R* = 0.751, *p* < 0.01. The clinical scale of the depth of sedation/anesthesia increased monotonically and positively with increasing propofol Ce concentration until LORP, revealing an increasing deepening of sedation/anesthesia.

### Multinomial logistic analysis

Regarding MLR, we choose the features which were better correlated with the Ce concentration of propofol and with aRASS, and features which we believe were useful (with a *P*_*k*_ > 0.700). The predictor features corresponding to these criteria were propofol Ce, and *V*_mean_, *V*_diff_, *f*_mean_, *f*_median_, *P*_bandwidth_ and spectral entropy, during the period *T*_1_, and *f*_mean_ and spectral entropy during the period *T*_2._

We tried to explore the effects of these variables by building the MLR model and then examined the results. To achieve this goal, we used SPSS software version 26, and calculated the MLR model with response variable and all explanatory variables to make the primary model.

The overall effectiveness of the model was assessed using Chi-squared statistics. The Chi-square value of 696.430 and its respective *p* value < 0.001 indicated a significant relationship between the depth of sedation/anesthesia scale and the set of features in the final model. Model performance (Nagelkerke *R*^2^) of the MLR for the combined features predicting aRASS level of sedation/anesthesia groups was 0.697. The overall predictive accuracy for the present model was 73.6%.

The likelihood ratio test shows the contribution of each feature to the model (Table 4).

**Table 4:** Likelihood ratio tests

		− Log likelihood	Chi-square	df	*p* value
Intercept		1000.646	16.418	5	0.006
Propofol Ce		1313.757	329.529	5	< 0.001
*T*_1_	*V*_mean_	993.034	8.806	5	0.117
	*f*_median_	1002.125	17.897	5	0.003
	*f*_median_	994.237	10.010	5	0.075
	*P*_bandwidth_	992.996	8.768	5	0.119
	Spectral entropy	1000.786	16.558	5	0.005
*T*_2_	*f*_mean_	994.827	10.599	5	0.060
	Spectral entropy	1001.268	17.040	5	0.004

*V*_mean_ mean amplitude, *f*_mean_   mean frequency, *f*_median_ median frequency, *P*_bandwidth_ power bandwidth; Ce  effect-site concentration

**Fig. 3:**
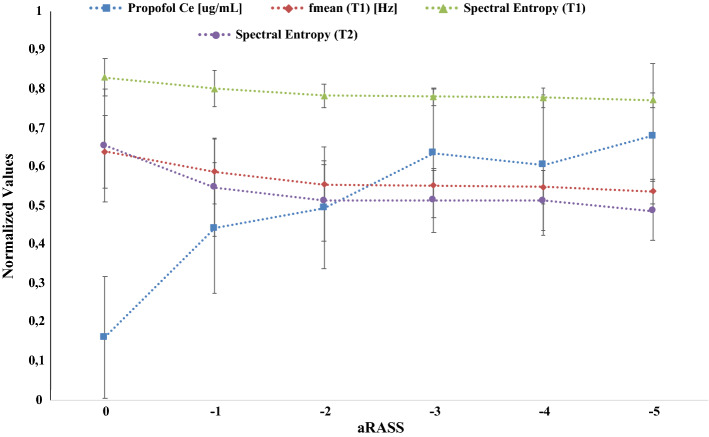
The adapted Richmond Agitation–Sedation Scale (aRASS) vs mean and standard deviation: (1) effect-site concentration (Ce) of propofol; (2) mean frequency (*f*_mean_ [Hz]) during the time window period *T*_1_; (3) spectral entropy during the time window period T_1;_ and (4) spectral entropy during the time window period *T*_2_

Propofol Ce_,_ *f*_mean_ (*T*_1_), spectral entropy (T_1_), and spectral entropy (*T*_2_) had a significant contribution (*p* < 0.05) to the model that predicts the aRASS’s level of sedation/anesthesia, but not *V*_mean_ (*T*_1_)_,_ *f*_median_ (*T*_1_)_,_ *P*_bandwidth_ (*T*_1_) or *f*_mean_ (*T*_2_). Figure 3 illustrates the relation between the aRASS score and the mean of the significant aforementioned features.

The fitted logistic model was

1 $$ \ln \left( {\frac{P}{1 - P}} \right) = - 339.206 + 2.476 \times {\text{Propofol Ce }} - 0.281 \times V_{{{\text{mean}}}} \left( {T_{1} } \right) - 5.422 \times { }f_{{{\text{mean}}}} \left( {T_{1} } \right) + 3.234 \times f_{{{\text{median}}}} \left( {{\text{T}}_{1} } \right) + 0.116 \times {\text{P}}_{{{\text{bandwith}}}} \left( {T_{1} } \right) + 1441.537 \times {\text{ Spectral entropy }}\left( {T_{1} } \right) + 0.074 \times { }f_{{{\text{mean}}}} \left( {T_{{2}} } \right) - 38.100 \times {\text{Spectral entropy }}\left( {T_{{2}} } \right), $$

where *P* is the estimated probability of unresponsiveness (i.e., aRASS = − 5).

## Discussion

In the present study, a standard electrical stimulus to evoke a blink reflex was used during the induction phase of general anesthesia with propofol and remifentanil to assess the relation between the recorded electromyogram and the level of sedation/anesthesia. The level of sedation was assessed every 6 s using an adapted version of the Richmond Agitation–Sedation Scale, entitled aRASS.

The electromyographic-derived features were extracted during 2 specific subsets of samples: *T*_1,_ from 10 to 25 ms, in which it was expected the first response of the blink reflex, *R*_1_, to be analyzed, and *T*_2_, from 25 to 200 ms, in which the second response of the blink reflex, *R*_2,_ was expected to be analyzed. The electromyographic-derived features in the time domain included the mean amplitude (*V*_mean_), and the difference between the maximum and the mean amplitude (*V*_diff_). Because *R*_2_ and *R*_1_ responses were abolished before LORP and, consequently, there was an insufficient number of data points, a frequency-domain analysis was also performed. The electromyographic-derived features in the frequency domain included the mean power (*P*_mean_), maximum power (*P*_max_), mean frequency (*f*_mean_), power at mean frequency (*P*_meanfreq_), median frequency (*f*_median),_ band power (*P*_band_), total power (*P*_total_), ratio between *P*_total_ and *f*_median_ (*P*_total_/*f*_median_), signal-to-noise (SNR), power bandwidth (*P*_bandwidth_) and spectral entropy. These variables were selected as they are the most useful and popular frequency-domain features for electromyography analysis both in clinical and engineering applications.

The ability of the electromyographic-derived features to distinguish different levels of aRASS was assessed using prediction probability analysis. Spectral entropy, *f*_mean_, *f*_median_ and *P*_bandwidth_ during the *T*_1_ time window period showed the best performance in detecting different levels of aRASS, as reflected by its higher *P*_*k*_ value, followed by spectral entropy and f_mean_ during the *T*_2_ time window period. A statistically significant correlation (*R* > 0.500) between aRASS (or propofol Ce concentration) and *f*_mean_ (*T*_1_), *f*_median_ (*T*_1_) and spectral entropy (*T*_1_) period was also found. At low anesthetic concentration, the EMG frequency was high, and it slowed down as the drugs concentrations increased. Spectral entropy considers both the overall signal variability characteristics, which are naturally related to the spectral content, and the signal’s complexity or irregularity [12]. For this reason, spectral entropy is known to be an excellent index to distinguish between consciousness and unconsciousness states during propofol anesthesia, even in the presence of burst suppression [13]. f_mean_ and f_median_ are frequently used as the gold standard tool to detect force in the target muscles using EMG signals [14, 15]. *P*_bandwidth_ is supposed to be a good indicator of changes in the EMG signal when certain frequencies are lost, as is the case of the R_1_ and R_2_ component of the blink reflex, which have a particular signature. The effectiveness of spectral entropy, *f*_mean,_ *f*_median_ and *P*_bandwidth_ to distinguish the different levels of aRASS, resulted by the inhibition of EMG activity by muscle relaxation, is presented and confirmed in this study.

Another finding was that aRASS scale was strongly correlated with estimated propofol Ce concentrations, indicating that the clinical scale aRASS increased monotonically and positively with increasing estimated propofol Ce concentrations until LORP. This revealed an increasing deepening of anesthesia. This finding is in line with those published by Mourisse and colleagues [10, 11] who have done a similar study using a different sedation scale and a different anesthetic protocol. In the work of Mourisse and colleagues, the group of patients received propofol in a stepwise deepening of anesthesia with different targets and only 2 min after reaching target effect-site concentrations, the blink reflexes, and depth of anesthesia scores were recorded. In our study, remifentanil infusion started with a Ce concentration target 2.5 μg/mL, and then patients received propofol at an infusion rate of 3.3 mL/kg/h, slowly and continuously, until LORP. Starting before the propofol infusion, the stimulation of the supraorbital nerve was recorded every 6 s in our study and, for this reason, our method had more data to precisely identify the amount of propofol in the endpoints of interest.

Only the features that were both useful for predicting the aRASS scale (*P*_*k*_ > 0.700) and correlated significantly (*R* > 0.500) with the propofol Ce concentration were used for the multinomial logistic regression model to predict LORP (defined as − 5 in the aRASS scale). The results of the multinomial analysis showed a reasonable performance of the model, explaining almost 70% of the aRASS variance. The effects and contributions of each feature were not the same: propofol Ce concentration, *f*_median_ (*T*_*1*_), spectral entropy during T_1_, and spectral entropy during T_2_ had a significant overall effect (*p* < 0.05) on the aRASS score, while *V*_mean_ (*T*_1_), *f*_median_ (*T*_1_) and *f*_mean_ (*T*_2_) did not. The overall predictive accuracy for the model was 73.6%, suggesting that it is useful to predict LORP. The availability of accurate models for predicting the drug effect in patients undergoing general anesthesia is an important factor in producing a personalized drug infusion [16]. Our developed model can be employed in model predictive control strategies for closed-loop anesthesia. This will help the anesthesiologists with the optimization of drug titration without overshoot and controlling the physiological functions. This automated system could also result in a reduction of the workload of the anesthesiologists.

A major drawback of this our blink reflex method is that it is dependent on a normal neuromuscular transmission. The degree of relaxation can be estimated by stimulating the facial nerve and assessing the evoked response of that part of the orbicularis oculi muscle. The effect of muscle relaxants on the inferior part of the orbicularis oculi is still not known. Also, the blink reflex method, while promising, may imply that the anesthesiologists are stimulating the patient while simultaneously attempting to induce unconsciousness, even though it is a much smaller stimulation than taping (the standard in clinical practice).

The multinomial regression model was applied to a small sample size of the unconsciousness states in this study. In particular, the small number of unconsciousness states could be the cause of a not so high performance in detecting LORP. Even so, the performance of our model is relatively good and therefore, we intend to implement our model for online estimation. However, preliminary studies should be conducted specially because of the computerization times the model can result.

## Conclusions

By analyzing the electrically evoked blink reflex during the induction of general anesthesia with propofol and remifentanil, we determined that there is enough relevant information to predict the state of unresponsiveness during the transition from consciousness to unconsciousness with a multinomial logistic model. A method that could allow for an automatic prediction/detection of LORP is a step forward for personalized medicine. With an accuracy of 73.6%, this model can help to greatly reduce the overwhelming variability observed between patients and avoid many cases of overdosing and associated risks. To our best knowledge, no studies have been conducted on LORP prediction using our approach.

Despite our results, it would be accurate if we had a larger sample size for applying the multinomial regression model or any other prediction model to analyze the relation between the aRASS and the EMG effect reflected in extracted features. Further research should investigate the impact of remifentanil on such a technique.

Nevertheless, our method of electromyographic recording of the electrically evoked blink reflex in patients submitted to general anesthesia and in the continuum to unconsciousness showed to be a possible method to continuously monitor the EMG, in awake, sedated or unconsciousness patients, during the onset to unconsciousness, and in a real scenario in which clinical anesthesia takes place every day. Patients also found the stimuli to be easily tolerable. Devices using this technique could turn this method into a clinical routine way of monitoring the transition to unconsciousness.

In the future, we intend to add other variables to feed the model, such as heart rate, blood pressure, and electroencephalogram. We plan to use those variables to build an adaptive model that deals simultaneously with the variabilities in the clinical response of the patients and with the drug interactions. Additionally, we plan to use different EMG equipment for more robustness.

## Methods

### Patients

Twenty-five patients, aged over 18 years (ASA I, II or III), scheduled for neurosurgical procedures participated in this study. They had no hemodynamic, respiratory or ophthalmic problems, and did not use analgesics, psychotropic or excessive alcohol consumptions. The Hospital Ethical Committee approved the study and all subjects gave informed written consent. No premedication was given. The study took place in a quiet, warm anesthetic induction room. Before the start of the study, the patients were prepared as usual for anesthesia (intravenous access, continuous electrocardiogram, pulse oximetry, and non-invasive blood pressure).

### Anesthetic protocol

Our standard practice for neurosurgical procedures consists of opioid-propofol anesthesia using a Target Controlled Infusion (TCI) system. In the operating room, after placement of standard monitor and an intravenous line in the dorsum of the hand, an infusion of a balanced electrolytic solution was started at 6 mL.kg^−1^.h^−1^. The anesthesiologist would then use a Fresenius Base Primea docking station (Fresenius-Kabi, Bad Homburg, Germany) to start a TCI of remifentanil (Minto PKPD model) [12, 13], at an effect-site concentration (Ce) target of 2.5 ng.mL^−1^. A bolus of 10 mg of lidocaine was administered locally to reduce the pain associated with propofol administration. One minute after the remifentanil pseudo-equilibration was achieved, baseline blinks were recorded and, then, an infusion of 1% propofol (Schnider PKPD model) [14] was started using a TCI enabled infusion system, in the TCI-View mode, at 3.3 mL.Kg^−1^.h^−1^ until LORP, determined by the anesthesiologist. This slow velocity of infusion during induction enabled a careful titration of the minimum amount of propofol required for loss of responsiveness. Once LORP was reached, the propofol infusion was stopped, the estimated propofol Ce concentration was noted and the TCI system was switched to effect-site TCI mode with a Ce of 75% of that at LORP. At this point, no additional analgesic/opioid medication was given during induction.

Propofol infusion was subsequently titrated to maintain BIS (BIS Vista™ monitor—Medtronic, Ireland) between 40 and 60. The study was terminated just before tracheal intubation.

### Data acquisition

**Fig. 4:**
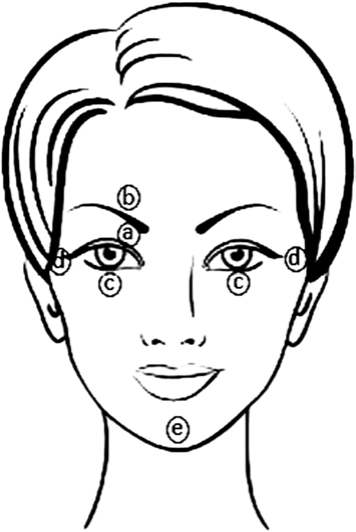
Electrode placement for blink reflex monitoring. Electrodes (**a**) (cathode) and (**b**) (anode) are electrodes used for electrical stimulation. Electrodes (**c**) (active) and (**d**) (reference) are silver disc recording electrodes. Electrode (**e**) is a ground electrode

Using the VikingQuest™ neurophysiological system (VikingQuest, Nicolet, WI, USA) the electromyographic stimulations and recordings were performed at a total sweep time of 200 ms with a sample rate of 10 kHz. A high-pass filter was applied with a cutoff frequency of 20 Hz. Before the induction of anesthesia and prior electrode application, all the patient’s head skin surfaces were cleaned with an exfoliant agent. Surface electrodes (1.4 cm^2^) coated with alcohol and conductive paste (electrode impedance < 8 kΩ) were applied to stimulate and record the electromyogram from the right orbicularis oculi muscle. The right supraorbital nerve was transcutaneously stimulated using a bipolar electrode with the cathode placed beneath the eyebrow over the supraorbital notch and the anode placed above the eyebrow (interelectrode distance 2 cm). The supraorbital nerve was electrically stimulated with a duration of 0.1 ms at 0.16 Hz. With regard to the electrode, the recording electrode was placed in the middle of the inferior rim of the orbit; the reference halfway on the eye–ear line and the ground electrode was placed on the cheek or on the shoulder of the patient (Fig. 4). The signals were stored in the VikingQuest™ software provided by the manufacturer. The raw data was exported to a personal computer to be treated and analyzed in MATLAB® 2019b (MathWorks, USA).

#### Assessment of levels of responsiveness

The level of sedation was assessed every six seconds using an adapted version of the Richmond Agitation–Sedation Scale (RASS) score [17], entitled aRASS. This scale is a modification of the RASS scale [17], with the addition of the corneal reflex endpoint, yielding an adapted Richmond Agitation–Sedation Scale (aRASS). A score of 0 corresponds to a fully awake and alert behavior, − 1 with a not fully alert behavior, but a sustained awakening to voice (eye-opening and eye contact), − 2 with a brief awakening to voice (eye-opening and eye contact, − 3 no response to voice, but movement or eye-opening to shaking and shouting, − 4 is no response after shaking and shouting, but still have the corneal reflex and − 5 is no corneal reflex. Loss of responsiveness (LORP), defined as − 5 in aRASS scale was evaluated using a drop of sterile water to the cornea, after aRASS reached a score of − 4. Hereafter, this evaluation was intercalated with electrical stimulations at 6 s intervals. If the eyes blinked concomitantly, the reflex was intact. If only one eye blinked, the reflex was impaired, and if neither eye blinked, the reflex was absent.

Baseline blink reflexes and evaluation of the aRASS scale were recorded several times before propofol was administered. Patients then received propofol, and at the same time four successive blink reflexes were elicited and recorded. A further four successive blink reflex stimuli were elicited after a 6-s interval and recorded continuously until LORP was reached.

#### Blink reflex parameters

The blink reflex neurophysiology and anatomy are reasonably well known [18]. The electromyography records of an electrically evoked blink reflex showed at least two components (*R*_1_ and *R*_2_ components). The first or early response (*R*_1_) is brief and occurs after a latency of approximately 10 ms on the side of stimulation [19]. The second response (*R*_2_) has a latency of approximately 30 ms, is bilateral, and more prolonged in time [19]. The *R*_2_ response causes the actual contraction of the orbicularis oculi muscle [19]. The optimal stimulus intensity was sought by gradually increasing the current until visual observation of the EMG showed that, in the presence of a visible *R*_1_ component, the *R*_2_ component reach its maximum amplitude [20].

**Fig. 5:**
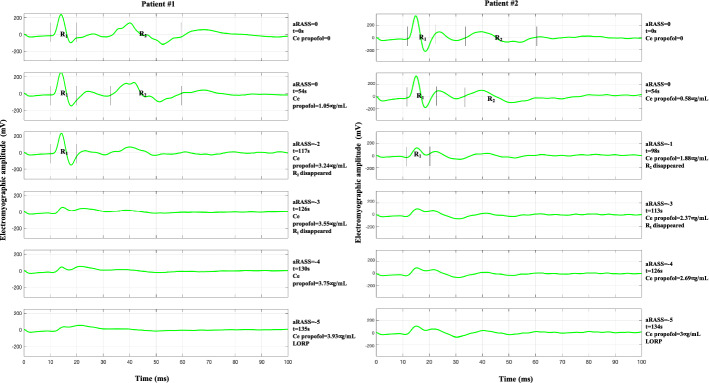
Examples of electromyographic records of the average from four consecutive blink reflex responses of **a** Patient #1: a male patient (60 years, 65 kg), and **b** Patient #2: a female patient (47 years, 64 kg) who participated in this study. Each row shows the effects of the increasing administration of propofol. In Patient #1, after 117 s of propofol infusion, the *R*_2_ component disappeared. *R*_1_ component was last seen after 126 s, loss of responsiveness (aRASS = − 5) occurred after 135 s after propofol infusion, at an effect-site concentration of 3.93 µ/mL. In Patient #2, after 98 s of propofol infusion, the *R*_2_ component disappeared.* R*_1_ component was last seen after 113 s, loss of responsiveness (aRASS = -5) occurred after 134 s after propofol infusion, at an effect-site concentration of 3.00 µ/mL

Figure 5, uppermost panels, illustrates the two components of a normal blink reflex. The averaged electromyographic records of the blink reflex were obtained from the four consecutive electrical stimulation of the supraorbital nerve (interstimulus interval 5 ms). The first rows showed baselines. Vertical lines marked the beginning and end of the individual components (*R*_1_ and *R*_2_), marked by an expert neurophysiologist at the end of the session, using the marker tool of the VikingQuest™ neurophysiological device. The right margins showed estimated propofol concentrations (µg/mL), clinical endpoints (loss of *R*_1_, loss of *R*_2_ and LORP), depth of sedation/anesthesia level (aRASS score), and the time since propofol started.

### Data analysis

Before proceeding with data analysis, we removed the DC component by subtracting the mean amplitude from the EMG signals. We then removed the best straight-fit line (in the least-squares sense) from the EMG signals with the detrend function of MATLAB®.

For each pre-processed EMG signal, we selected two specific subsets of samples: *T*_1,_ from 10 to 25 ms, in which it is expected R_1_ to be analyzed, and *T*_2_, from 25 to 200 ms, in which *R*_2_ is expected to be analyzed.

Data from each subset was analyzed in both time and frequency domain. In the time domain, after rectifying and normalizing each subset of data, the following features were extracted: mean amplitude (*V*_mean_), and the difference between the maximum (equal to 1) and the mean amplitudes (*V*_diff_).

Because *R*_2_ and *R*_1_ responses were abolished before LORP and, consequently, there was an insufficient number of data points, a frequency-domain analysis was also performed. A non-parametric Fast Fourier Transform (FFT) using Welch's modified periodogram was used to extract functions from the frequency domain. EMG was windowed with a Hamming window and the modified periodogram was calculated for each time period. The Power Spectrum Density (PSD) was estimated by averaging over all resulting periodograms. For this purpose, MATLAB® was used. The following features from the frequency domain were extracted:

- Mean power (*P*_mean_ [dB]): the arithmetic mean value of the PSD estimate;
- Maximum power (*P*_max_ [dB]): the maximum value of the PSD estimate;
- Mean frequency (*f*_mean_ [Hz]): the average frequency which is calculated as the sum of product of the power spectrum of the PSD estimate and the frequency divided by the total sum of the power spectrum [21].
- Power at *f*_mean_ (*P*_meanfreq_ [dB]): the value of the PSD estimate corresponding to the f_mean_.
- Median frequency (*f*_median_ [Hz]): the frequency at which the power spectrum of the PSD estimate is divided into two regions with equal areas;
- Band power (*P*_band_ [dB]): the sum of the PSD estimate values within the frequency range 30–120 Hz;
- Total power (*P*_total_ [dB]): the sum of all the PSD estimate values;
- Ratio between *P*_total_ and *f*_median_ (*P*_total_/*f*_median_ [dB/Hz]);
- Signal-to-noise ratio (SNR [dB]): the ratio of the PSD of the signal (meaningful information) with respect to the power of the background noise;
- Power bandwidth (*P*_bandwidth_ [dB]): the 3-dB (half-power) bandwidth of the PSD estimate;
- Spectral entropy: The Shannon entropy of the PSD estimate, i.e., the evaluation of the shape of the PSD estimate [22].

#### Prediction probability analysis

The capacity of the extracted features to detect the different sedation/anesthetic levels reflected in the numerical scale of anesthesia, aRASS was evaluated using prediction probability (*P*_*k*_) statistics by correlating the parameter values during the two different study periods with the numerical scale.

To calculate *P*_*k*_, we used a user-friendly program, the *P*_*k*_ Tool version 3.0, which included a context help, and colleagues [23] kindly provided the software and developed the algorithm according to the original [24]. A value of *P*_*k*_ = 0.5 means that the indicator/feature correctly predicts the sedation/anesthetic states only 50% of the time, i.e., no better than a 50:50 chance. A value of *P*_*k*_ = 1 means that the indicator/feature predicts the aRASS correctly 100% of the time. *P*_*k*_ was calculated using pooled data from all patients.

#### Multinomial logistic analysis

The relation between the aRASS level of sedation/anesthesia and the EMG effect reflected in each extracted feature was modeled with a multinomial logistic regression (MLR) using a multiple predictor features defined by:

2 $$ ln\frac{P}{1 - P} = \beta_{0} + \mathop \sum \limits_{k = 1}^{n} \beta_{k} X_{k} , $$

where *P* represents an outcome (or dependent variable) as a probability, *β*_0_…*β*_n_ represent the constant of the coefficients of the regression model, and *Χ*_1_…*Χ*_n_ represent the predictor (or independent) variables [25].

### Statistics

The Kolmogorov–Smirnov test was used to test for normal distribution. Paired samples t-test or Wilcoxon signed-rank test was used to compare the means of related data. Correlation analysis was performed using the Pearson *R* test for normally distributed data or the Spearman rank R when data were not normally distributed. Data were presented as mean along with standard deviation (SD) and 95% confidence interval (CI), unless stated otherwise, in our logistic regression analysis.

In the same experimental session of each subject, we evaluated our regression models using mean values, otherwise, the variance of each predictor variable would be computed as much lower. $P\text{ value} \, \text{<} \, \text{0.05}$ was considered to be statistically significant.

## Data Availability

All data generated or analyzed during this study are included in this published article.

## Abbreviations

aRASS: Adapted Richmond Agitation–Sedation Scale
BIS: Bispectral Index; Ce: Effect-site concentration
EMG: Electromyography
*f*_mean_: The average frequency which is calculated as the sum of product of the power spectrum of the PSD estimate and the frequency divided by the total sum of the power spectrum
*f*_median_: Frequency at which the power spectrum of the PSD estimate is divided into two regions with equal areas
*P*_max_: The maximum value of the PSD estimate
LORP: Loss of responsiveness
LOR_1_: Loss of R_1_
LOR_2_: Loss of R_2_
MLR: Multinomial logistic regression
*P*_band_: The sum of the PSD estimate values within the frequency range 30–120 Hz
*P*_bandwidth_: The 3-dB (half-power) bandwidth of the PSD estimate
*P*_bandwidthband_: The 3-dB (half-power) bandwidth of the PSD estimate within the resolution bandwidth 30–120 Hz
*P*_*k*_: Probability prediction
PKPD: Pharmacokinetic/pharmacodynamic
*P*_max_: The maximum value of the PSD estimate
*P*_mean_: The arithmetic mean value of the PSD estimate
*P*_meanfreq_: The value of the PSD estimate corresponding to the f_mean_
PSD: Power spectrum density
*P*_total_: The sum of all the PSD estimate values
*P*_total/_*f*_median_: Ratio between P_total_ and f_median_
*R*_1_: First component of the blink reflex
*R*_2_: Second component of the blink reflex
RASS: Richmond Agitation–Sedation Scale
SNR: Signal-to-noise ratio
*T*_1_: Time window 1 (from 10 to 25 ms)
*T*_2_: Time window 2 (from 25 to 200 ms)
*V*_max_: Maximum amplitude of the EMG signal
*V*_mean_: Mean amplitude of the EMG signal
*V*_diff_: Difference between the maximum and the mean amplitudes
